# Supplementary material for: Gemykibivirus detection in acute encephalitis patients from Nepal
Source: mSphere. 2024 Jun 21;9(7):e00219-24. doi: 10.1128/msphere.00219-24 (PMC11287993; doi:10.1128/msphere.00219-24)
Supplement: Table S1 — Primers used for PCR. [file msphere.00219-24-s0001.docx]

Table S1: Primers used for PCR

| Primer name | Sequence 5' to 3' | Binding Sites |
| --- | --- | --- |
| Gemy8917-9967F | TCAACGACCTCTGATACATACC | 429 <- 451 |
| Gemy8917-9967R | TAGATGAGTTCCACCATCAGC | 2034 -> 2055 |
| Gemi1625confirmR | GGCATTGCAATTATGGCTTATGGT | 88 -> 113 |
| Gemi1625confirmF | GGCAAGAAACTCGTCCACTGGG | 1194 <- 1215 |
| Gemy9967-8917R | TCATAATCTGCTCCGTGTTCCT | 1133 -> 1154 |
| GemykibiTM_9967F | GGTCAGAGCCTAGTGTTGTATG | 1419 <- 1440 |
| Gemy1xgenomeF | TTAATCGATCTAGAGGATCCTTGTTAGATATCCATATGGCGG | 1160 -> 1187 |
| Gemy1xgenomeR | TTAGTAATGGGCCCGGATCCACGAGAGGAACACG | 1146 <- 1165 |
